# Design of a Consensus Derived Synoptic Operative Report for Thyroid Surgery

**DOI:** 10.1002/wjs.12673

**Published:** 2025-06-20

**Authors:** Edwina C. Moore, Carolyn Garner, Jonathan Serpell, Mark Sywak, Jeremy Millar, Liane Ioannou, Rasa Ruseckaite, Susannah Ahern

**Affiliations:** ^1^ Endocrine Surgery Peninsula Private Hospital Melbourne Australia; ^2^ Endocrine and Oncologic Surgical Associates Mariposa Surgery Centre Corinth Texas USA; ^3^ Breast, Endocrine and General Surgery Alfred Health Monash University Melbourne Australia; ^4^ Northern Clinical School Sydney Medical School Faculty of Medicine and Health University of Sydney Sydney Australia; ^5^ Endocrine Surgical Unit Royal North Shore Hospital St Leonards Australia; ^6^ Radiation Oncology Alfred Health Monash University Melbourne Australia; ^7^ School of Public Health and Preventive Medicine Monash University Melbourne Australia

**Keywords:** operative reports, synoptic, thyroid surgery

## Abstract

**Background:**

The aim of this study was to develop a template for a synoptic operative report (SOR), for thyroid surgical data, and improve interdisciplinary communication and collaboration.

**Methods:**

Twenty‐five expert endocrine surgeons, endocrinologists, surgical oncology, and ear‐nose‐and‐throat surgeons were invited to participate in a modified Delphi process. Initial domains for consideration were determined from literature review and were compatible with domains previously endorsed by the American Thyroid Association. Consensus for individual variables was predefined as mean > 70th percentile and a low disagreement index (< 1.0). An academic working party reviewed the indeterminate domains to reach a final consensus.

**Results:**

Of 25 invited, 24 participated in the study. Two rounds of questionnaires were conducted. Consensus was reached for 39 data elements across five domains and were included in the final SOR template. A further eight data elements were shortlisted for inclusion in a secondary list of desirable but optional data elements.

**Conclusion:**

We developed a consensus‐derived SOR for thyroid surgery. Future studies are required to review the uptake and utility of this template in a clinical setting. There is a literary void regarding the role of SOR for parathyroid and adrenal surgery and this could be approached with similar methodology.

## Graphical abstract

The aim of this study was to develop a template for a synoptic operative report (SOR), for thyroid surgical data, and improve interdisciplinary communication and collaboration. Twenty‐five expert endocrine surgeons, endocrinologists, surgical oncology, and ear‐nose‐and‐throat surgeons were invited to participate in a modified Delphi process. Initial domains for consideration were determined from literature review and were compatible with domains previously endorsed by the American Thyroid Association.

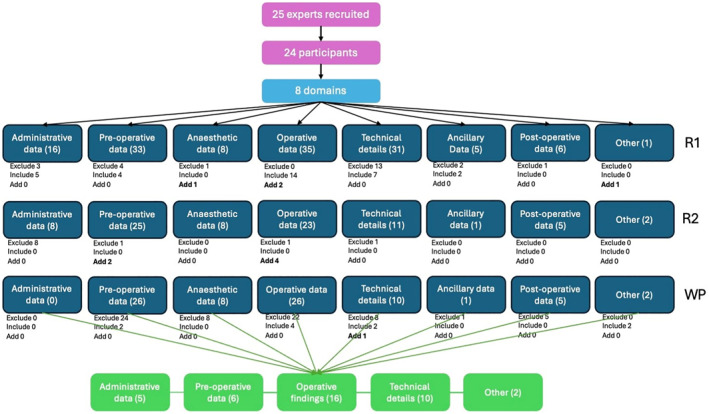

## 1 Introduction

Medical communication is underscored by accurate, contemporaneous, and relevant clinical notes [1]. Effective communication is the fundamental instrument in reducing adverse events and improving patient outcomes [2]. In surgery, it is universally accepted that an operative report must be prepared after any procedure is undertaken. The purpose of an operative report is to record critical findings and to disseminate fundamental surgical information to other surgeons, physicians, and healthcare providers, relevant for ongoing management.

Historically, a narrative operative report (NOR) has been standard practice for surgical documentation. This comprises either a verbal or computer‐based dictation in a narrative style. However, the ability of NOR to succinctly and reliably capture relevant surgical information varies widely. Several studies have shown that an NOR may omit intraoperative details in up to half of cases [3, 4]. Furthermore, failure to document specific operative details may be retrospectively interpreted as a negative finding rather than a true omission [5].

Synoptic reporting is a process for reporting specific data elements in a specific format or against a checklist. In many aspects of healthcare, synoptic reporting is increasingly utilized with excellent outcomes in terms of cost, efficiency, reproducibility, accuracy, and quality [6]. Synoptic pathology reporting has been associated with vast improvements in the overall clarity and completeness of reporting and has gained widespread international adaptation [7]. Additionally, synoptic reporting enables standardized data collection thereby enhancing surgical research and collaboration [8].

In 2014, a Canadian pilot project examined the potential of a digital synoptic reporting system for breast, colorectal, ovarian, and head/neck operations. They demonstrated improved efficiency using synoptic reports, an overall positive experience by surgeons, and more comprehensive surgical documentation [9].

Synoptic operative reporting (SOR) is particularly compatible for reporting in thyroid surgery, which has several well‐defined sequential steps to complete the procedure satisfactorily. For patients who have multiple operations in the same anatomical area, for example, patients with hereditary endocrine tumors, SOR provides considerable benefits.

SOR has the potential to transform surgical documentation and communication. It allows for the accurate collection and comparison of data affecting patient outcomes and improves patient safety by reducing communication errors. In addition to capturing surgical data, templates may be reviewed to allow assessment of adherence to clinical guidelines and safety procedures [10, 11]. Ultimately, the data obtained in the SOR will be reproducible and therefore codable. There may also be scope for enhanced digital data computation and export.

The primary aim of this study was to develop a consensus‐derived template for SOR for thyroid surgery.

## 2 Methods

Twenty‐five select members of Australia and New Zealand Endocrine Surgeons (ANZES), endocrinologists and international endocrine, surgical oncology, and ear/nose and throat (ENT) surgeons were invited to participate in the study via an expression of interest campaign. An introductory email with details about the project was sent by the primary author. Experts were selected based on their breadth of experience with thyroid pathology, although being mindful to create a panel with balanced age, sex, and geographic representation.

The primary author and a small academic working party (WP) defined a preliminary set of data elements via literature review and expert opinion. The WP included four surgeons, a nonclinical academic cancer specialist with expertise in Delphi processes and developing consensus guidelines and a chairperson. The data elements were associated with umbrella domains suggested by the American Thyroid Association's (ATA) “Statement on the Essential Elements of Interdisciplinary Communication of Perioperative Information for Patients Undergoing Thyroid Cancer Surgery” [12], including domains of administrative details, preoperative data, anesthetic details, operative findings, technical details, ancillary data, postoperative data, and other unclassified information. The data elements formed the basis of the survey.

Participants were required to complete two rounds of an anonymous electronic survey, adhering to published guidelines on the Delphi survey methodology [13]. They were asked to assess potential data elements (within a particular domain), worthy of inclusion in a SOR for thyroid surgery. For example, “*how important is documenting the patient* ***medical record number (MRN)*** *in the SOR?*”—is associated with the umbrella domain of administrative details and the data element of medical record number (MRN). Participants were asked to rate each data element (e.g., MRN) using a Likert scale (1 of low importance to 10 of high importance) in the context of inclusion within a SOR. Reminder emails were sent every 2 weeks until the surveys were completed. The surveys were conducted on the Qualtrics platform (Provo, Utah, USA).

The first round was conducted to determine which data elements (e.g., MRN, date of birth, and email) should be included in the SOR. Consensus for inclusion of particular data elements was predetermined: if the mean score is > 6.3 (70th percentile) and had a low disagreement index (DI < 1). Consensus for exclusion was if the mean was < 2.7 (30th percentile). The disagreement index was calculated as the interpercentile range (IPR) divided by the interpercentile range adjusted for symmetry (IPRAS) [14]. A second round was undertaken to further evaluate data elements, which did not achieve consensus (i.e., > 30 and < 70th percentiles and/or high disagreement index), or were suggested as a new domain in the first‐round survey. The assessment criteria were individual item importance and feasibility.

**FIGURE 1:**
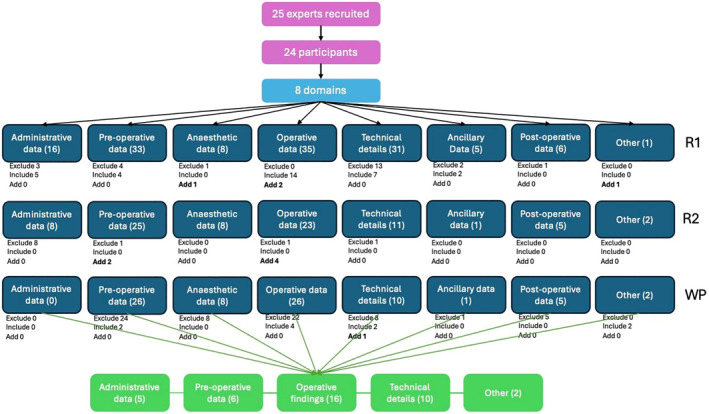
Flow diagram of the modified Delphi process to establish a consensus‐derived template for SOR for thyroid surgery. R1 = round 1, R2 = round 2, and WP = working party.

On completion of the surveys, all the domains that achieved consensus and the outstanding indeterminate domains were discussed amongst the WP. The shortlisted data elements were individually discussed and further assessed in terms of clinical relevance. With the aim of developing a practical template, time taken to complete the prospective SOR was also considered and the maximum number of data elements to be included was capped at 40. The WP developed two datasets: core (tier 1) and optional (tier 2). The SOR template was prepared by the first author based on the core dataset and approved by the WP (Figure 1).

The project was approved by the Alfred Health Human Research Ethics Committee prior to commencement (project ID39/24).

## 3 Results

### 3.1 Participant Information

Of the 25 invited experts, 24 completed each round of the survey (96% response rate). The overwhelming majority were endocrine surgeons (83%), followed by endocrinologists (8%), ENT surgeons (4%), and surgical oncologists (4%). The level of expertise was very high. More than half of participants had > 15 years of independent practice postfellowship. A mixed academic and private practice was the most common practice type (58%). Overall, two thirds of participants were Australian. The most widely represented Australian state was Victoria (42%), followed by NSW (8%). Table 1 displays the overall characteristics of the participants.

**TABLE 1:** Summary of participant characteristics.

	Number of participants (*n* = 24)	Percentage (%)
Sex (female)	11	45.8
Level of experience (years postfellowship)		
< 5	2	8
5–15	8	33
15–30	8	33
> 30	6	25
Subspecialty		
Endocrine surgery	20	83
Ear, nose, and throat surgery	1	4
Endocrinology	2	8
Surgical oncology	1	4
Type of practice		
Academic/University affiliated	7	29
Mixed	14	58
Nonacademic/Private	3	13
Location of practice		
International	7	29
Australia	17	61
Victoria	10	42
New South Wales	2	8
Queensland	1	4
Western Australia	1	4
South Australia	1	4
Tasmania	2	8

### 3.2 Delphi Procedure

In the first round, the survey questions related to 135 data elements across 8 domains. Of these, 32 (24%) reached consensus for inclusion in the SOR template. 24 items (18%) were excluded, 79 items (58%) were indeterminate, and 4 were suggested as new for consideration. The second round reviewed the remaining 83 items (79 + 4). Of these, 11 were excluded, no further were included, and 6 new data items were suggested comprising a total dataset of 78 (Table 2).

**TABLE 2:** Initial survey domains and number of associated data elements that were reviewed at various stages in the modified Delphi.

Survey domain	Round 1 *n* = 135	Round 2 *n* = 83	Working party *n* = 78	Final template
Administrative data	16	8	0	5
e.g., MRN				
Preoperative data	33	25	26	5 (6^a^)
e.g., preoperative ultrasound				
Anesthetic details	8	8	8	0
e.g., pressure care				
Operative findings	35	23	26	13 (16^a^)
e.g., RLN identified				
Technical details	31	11	10	5 (10^a^)
e.g., presence of residual thyroid				
Ancillary data	5	1	1	0
e.g., use of autofluorescence				
Postoperative data	6	5	5	0
e.g., thyroxine dose				
Other	1	2	2	2
e.g., blank space for sketch				
**Achieved consensus to include**	32	0	+8	30 (39^a^)
**Achieved consensus to exclude**	−24	−11	−70	—
**Suggested as new**	+4	+6	+1	—

Abbreviations: MRN = medical record number, *n* = total number of data elements reviewed, and RLN = recurrent laryngeal nerve.

^a^ Number if all binary questions are answered “yes.”

### 3.3 Post‐Delphi Working Party Review

Following the Delphi process, the WP reviewed the outstanding 78 data elements. In keeping with the aim of the project and clinical feasibility [15], they eliminated data items where similar information was obtainable elsewhere within the medical record (duplicate data) and focused on including unique data elements only (e.g., only obtainable from surgery). This reduced the number of indeterminate data elements to 40.

The WP eliminated a further 30 data elements based on the items being considered too subjective, irrelevant, and low likelihood of being regularly observed or abnormal in thyroid surgery. A new subdomain was added that included specific items regarding neural monitoring. In addition, the WP opted to override two data elements, which had already reached consensus (presence of abnormal lymph nodes and burden of lymph node disease), due to concerns about subjectivity of assessment. A further two were downgraded from tier 1 to tier 2 out of concern regarding reproducibility (need for frozen section and description of primary nodule). In addition, the WP recommended inclusion of a novel data element (cervical plexus blockade), as this is important for postoperative patient assessment and is not recorded elsewhere in the medical history. Ultimately, the WP agreed to include an additional 10 data elements in the template.

**FIGURE 2:**
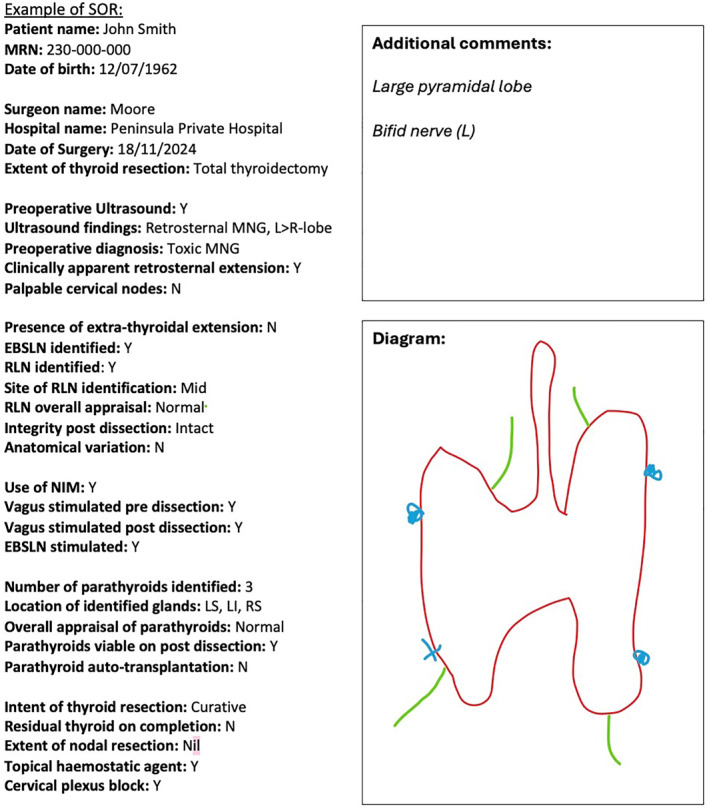
Example of completed SOR for thyroid surgery.

Following the Delphi procedure and WP discussion, the template for the SOR included five domains and up to a maximum of 39 data elements (if all the binary questions are answered “yes”). The tier 2 dataset included a further eight optional data elements (Figure 2; Tables 3 and 4).

**TABLE 3:** Template for SOR.

Administrative data	Patient details	Patient name	Free text
		MRN	Number
		Date of birth	DD/MM/YYYY
	Provider details	Surgeon name	Free text
	Hospital details	Hospital name	Free text
Preoperative data	Nonoperative details	Date of surgery	DD/MM/YYYY
		Preoperative diagnosis	Free text
		Preoperative ultrasound	Y/N
		–*If yes*	
		Ultrasound findings	Free text
	Clinical red flags	Clinically apparent retrosternal extension	Y/N
		Palpable cervical nodes	Y/N
Operative findings	General	Presence of extra‐thyroidal extension	Y/N
		EBSLN identified	Y/N
	RLN	RLN identified	Y/N
		Site of identification	High, mid, and low
		Overall appraisal of RLN	Normal, small, and inflamed
		Integrity post dissection	Intact/Compromised
		Anatomical variations	Y/N
	Neural monitoring	Use of intraoperative neural monitoring	Y/N
		–*If yes*	
		Vagus stimulated pre dissection	Y/N
		Vagus stimulated post dissection	Y/N
		EBSLN stimulated	Y/N
	Parathyroid	Number identified	0, 1, 2, 3, 4, and > 4
		Location of identified glands	LS, LI, RS, and RI
		Overall appraisal of parathyroids	Normal/Abnormal
		Viable post dissection	Y/N
		Need for parathyroid auto‐transplantation	Y/N
Technical details	Thyroid	Extent of thyroid resection	Total thyroidectomy
			Left hemithyroidectomy
			Right hemithyroidectomy
			Completion thyroidectomy
			Isthmusectomy
			Other
		Intent of thyroid resection	Curative, palliative
		Residual thyroid on completion	Y/N
		–*If yes*	
		Reason for residual thyroid	Preserve parathyroid
			Preserve nerve
			Tracheal invasion
			Other
		Topical hemostatic agent	Y/N
		Cervical plexus block	Y/N
	Nodes	Lymph node dissection	Y/N
		–*If yes*	
		Intent of nodal dissection	Curative/Palliative
		Extent of nodal dissection	Central/Lateral
		Nodal stations resected	I, II, III, IV, V, VI, and VII
		Laterality of nodal resection	Left, right, and bilateral
Other	Diagram	Blank space for sketch	
	Comments	Blank space to write additional details	

Abbreviations: EBSLN = external branch superior laryngeal nerve, MRN = medical record number, NIM = nerve integrity monitor, and RLN = recurrent laryngeal nerve.

**TABLE 4:** Tier 2 complementary data elements.

Operative findings	General	Tracheal invasion	Y/N
		Description of primary nodule	Free text
		Need for frozen section	Y/N
Technical details	Thyroid	Approach to straps	Preserve/Divide
		Use of Valsalva	Y/N
		Skin closure	Suture/Glue/Other
Other	Ancillary procedures	Type of ancillary tool	PT‐eye
			Autofluorescence
			Other
		Findings	Free text

Abbreviation: PT = parathyroid.

## 4 Discussion

We have developed a consensus‐derived template for SOR in thyroid surgery. This includes 39 data elements (tier 1). A further eight data elements were shortlisted as optional (tier 2). To the best of our knowledge, this is the first thyroid specific template for SOR with multicentric, international, and interdisciplinary input.

Synoptic reports have been successfully used in healthcare for several decades, for example, anatomical pathology for cancer [7, 16, 17], endoscopy [18], imaging [19, 20, 21, 22], and surgery (gynecological [23, 24, 25], pediatrics [26], colorectal [27, 28, 29, 30, 31], melanoma [32], breast [33, 34], upper gastrointestinal [35, 36, 37, 38, 39, 40], emergency general surgery [41], and thoracics [42]). In endocrine surgery, the use of SOR has evolved more slowly. In 2009, Chambers et al. presented the development of the first web‐based synoptic reporting system (WebSMR) for thyroidectomy. This included 46 mandatory data fields with drop down menus and comment boxes. They demonstrated that the WebSMR was associated with significant improvement in the completeness and accuracy of operative reporting at their institution [43]. In 2011, Iyer et al. developed a thyroid specific e‐form for surgical reports. This included 32 data elements and was based on the Caisis data management system for cancer. They demonstrated rapid completion of the e‐form but inferior completeness [44]. More recently the American College of Surgeons in partnership with the Commission on Cancer (CoC), launched new operative accreditation standards [45] including synoptic documentation of particular data elements in operative reports for an increasing number of surgical subspecialties (e.g., breast, melanoma, and rectal). This has been a launchpad for SOR for many operations.

It is important to define the primary purpose of a SOR, which is to record key surgical data. It should be easy to complete and widely accessible [46], although retaining a high level of clinical detail and potential scope for individuality [10, 11, 47, 48]. This is essential for patient care and future decision‐making. For patients with cancer and others requiring multiple operations in the same anatomical site, a clear and comprehensive record of surgical data cannot be overemphasized [49, 50]. Secondary roles of the SOR may include quality improvement [23, 40, 51, 52], research [30], education, and medicolegal evidence. The advantages of SOR over NOR include enhanced quality, efficiency, legibility, lower cost, and capacity for data sharing, for example, for clinical audit, medical billing, and multicenter research.

The SOR is not designed to be a synoptic overview of the entire clinical scenario. As much as practically possible, duplication of data from within the medical record should be avoided. In its present format, this template is a hard copy document, which can be printed and completed manually; however in the future, it could be converted into a hybrid application program (web/mobile app). Specialty society endorsement would inevitably enhance clinician uptake. The nonidentifiable surgical data captured within the SOR could also ultimately form the foundation of a self‐servicing surgical registry. This could facilitate large volume, multicenter, and real‐world data analysis and identification of variation in practice patterns.

An interesting concept, that is, rarely reviewed is charting by exception. In healthcare, among other industries, there is a tendency to document every step of a procedure, even when it has minimal clinical relevance, for example, type of prep used and size of suture. Charting by exception means to only record the details, which are atypical, for example, bifid nerve. Although this may enable a much shorter SOR and therefore ease of use, the working party felt that there is too much variability in technique and this approach would not be widely accepted. Furthermore, it would unacceptable in the event of a malpractice lawsuit, whereby the operative report is significantly scrutinized.

There are several limitations of this project, which should be acknowledged. First, only 24 experts participated in the Delphi, whereas there are hundreds of physicians involved in the care of thyroid patients in Australia and internationally. The appropriate number of participants in a modified Delphi is variable [53]. We attempted to mitigate bias by having experts from multiple centers, with variable levels of experience, mixed practice type, and specialty training. It is possible that a larger Delphi panel may have achieved a greater proportion of consensus items, requiring less involvement by the WP; however, this cannot be assumed. Secondly, none of the participants were from a European institution and we acknowledge a lack of diversity in this regard. However, several of the participants either completed training or have practiced within a European network. Finally, the WP eliminated the greatest number of indeterminate data elements and may be criticized for being too stringent. On balance, it is more reasonable to exclude items if consensus is not reached rather than include items that were not agreed to by a majority of participants. Presumably, the lack of consensus relates to variation in areas of clinical practice where there are no guidelines or recommendations. Ultimately, the aim of the WP was to create a workable template with broad appeal. In an attempt to minimize discontent amongst users, they also created a second list (tier 2) of optional data elements and the template includes a space for narrative comments.

## 5 Conclusion

Despite universal support for accurate recording of surgical data, there is no standardized format or terminology for operative reporting in endocrine surgery in Australia and further afield. In this study, we developed a consensus‐derived template for SOR for thyroid surgery. We have balanced the need to record meaningful data elements, with the demands of real‐world clinical practice (time pressures and resource constraints) and surgical flair. We have developed an open baseline set of data elements that we hope can to act as a minimal standard across a wide number of institutional settings, while at the same time providing a foundation for quality improvement and the potential for collaborative research.

## Author Contributions

**Edwina C. Moore:** conceptualization, writing – original draft, methodology, writing – review and editing, formal analysis, project administration. **Carolyn Garner:** writing – review and editing, data curation. **Jonathan Serpell:** data curation, supervision, writing – review and editing. **Mark Sywak:** writing – review and editing, data curation. **Jeremy Millar:** writing – review and editing, data curation. **Liane Ioannou:** methodology, writing – review and editing, supervision. **Rasa Ruseckaite:** writing – review and editing, supervision, methodology. **Susannah Ahern:** methodology, writing – review and editing, data curation, project administration, supervision, resources.

## Conflicts of Interest

The authors declare no conflicts of interest.

## Data Availability

The data that support the findings of this study are available from the corresponding author upon reasonable request.
